# The Role of Emotional Service Expectation Toward Perceived Quality and Satisfaction: Moderating Effects of Deep Acting and Surface Acting

**DOI:** 10.3389/fpsyg.2019.00321

**Published:** 2019-03-12

**Authors:** Ji Youn Jeong, Jungkun Park, Hyowon Hyun

**Affiliations:** ^1^School of Ecological Environment and Eco-tourism, Kyungpook National University, Sangju, South Korea; ^2^School of Business, Hanyang University, Seoul, South Korea

**Keywords:** emotional service, customer expectation, satisfaction, emotional labor, deep/surface acting

## Abstract

A conceptual model articulating the nature of customer expectations and satisfaction over services was proposed with emotional factors. Five propositions about consumer emotional service expectations as a primary antecedent toward confirmation, perceived quality, and satisfaction were provided. As moderators, two dimensions of consumer detection of emotional labor (i.e., detecting deep acting and surface acting) were imposed on each of the relationships. Evidence demonstrated the roles of emotional service expectation in service confirmation and satisfaction. The moderating effects of consumer detections of employees’ emotional strategies were limited to the relationship between emotional service expectation and confirmation; its relationship was weakened by detections of surface acting while the other relationships were not moderated by detections of deep nor surface acting. Structural equation modeling analyses were conducted using online survey data targeting consumers in the hotel industry.

## Introduction

Having motivated employees who proactively and professionally engage in customer interactions is undoubtedly critical to service firm’s success (Menguc et al., 2013; Schepers et al., 2016). In the hospitality industry, employees are deeply involved in everyday interactions with customers, such interactions being regarded as essential to the delivery of quality service experiences to customers (Farrell and Oczkowski, 2009). As a result, establishing effective strategies for employee–customer interactions, is recognized as the most important step in enhancing firms’ competitiveness (Lam et al., 2018). Indeed, huge resources are invested in training employees to enhance their interaction performance in the hospitality industry (Schepers et al., 2016).

Some researchers argue that service components are categorized into two attributes: (i) the core attributes that consist of functional quality factors; and (ii) the relational attributes that describe the interpersonally recognized factors during the service delivery (Babin et al., 2004). On the other hand, other researchers suggest that such a classification is artificial and fuzzy because the nature of services involves intangible and experiential consumption (Gaur et al., 2014; Koenig-Lewis and Palmer, 2014). They argue that factors perceived interpersonally by customers can be a core factor that significantly influences service quality, increasing consumer satisfaction and loyalty behaviors. These conflicting arguments lead to a main question of this study: does employees’ emotional service delivery during customer interactions, that is one of the relational attributes, significantly influence service outcomes, such as service confirmation, quality perceptions, and satisfaction? The second research question is as follows: if so, do different detections of employees’ emotional service strategies lead to different customer reactions? Typically, service employees are expected to express positive emotions and to suppress negative emotions in their interactions with customers (Diefendorff and Richard, 2003), but it is inherently impossible always to feel *genuine* positive emotions for customers (Groth et al., 2009). Consequently, employees attempt to utilize surface acting, faking a positive emotional display, to comply with their job requirements and meet customer needs for positive emotional services (Hochschild, 1983; Lam et al., 2018). However, customers may detect surface acting, and show less positive responses (i.e., service satisfaction and perceived quality) than when detecting deep acting (Lam et al., 2018).

To address these two research questions, we developed a theoretical model consisting of customer emotional expectations, overall confirmation, perceived quality, satisfaction, and two moderators of consumer detection of deep acting and surface acting. Affective elements have been well-reported in relation to the expectation – performance discrepancy link (Burns and Neisner, 2006) and customer satisfaction (Oliver, 1980). Nevertheless, the relationships between customer’s *emotional* service expectations and consumer satisfaction have not been fully addressed in the service literature. Furthermore, it has not yet been concluded how customer detections of employees’ emotional service strategies influence such relationships among emotional expectation, service quality perception, and satisfaction (Groth et al., 2009). Thus, this study would fill the void in the literature by addressing emotional service expectation and customer detection of the emotional display strategies in the hospitality service context.

## Literature Review and Hypotheses

### Emotional Service Expectation and Confirmation

Emotional labor can be defined as service employees’ efforts to demonstrate and express desired emotions at work by managing feelings (Hochschild, 1983; Ashforth and Humphrey, 1993; Grandey, 2000). For example, Hochschild (1983) defined emotional labor as “the management of feelings to create a publicly observable facial and bodily display” which is “sold for wage and therefore has exchange value” (p. 7). Similarly, Grandey (2000, p. 97) defined emotional labor as the “process of regulating both feelings and expressions for the organizational goals.” Previous literature has indicated critical roles of emotional labor in the service context. Tsai (2001) stated that employees’ emotional services are all part of the service itself. Hochschild (1983) also pointed out that “the emotional style of offering the service is part of the service itself” (p. 5). The importance of emotional services in confirming customer expectation is unanimously accepted among scholars and practitioners in the service field (Park et al., 2018).

The notion of *confirmation* has been explained in relation to *expectation*, which is defined as belief probabilities of the consequences of an event (Roch and Poister, 2006). Burgoon and Walther (1990, p. 236) identified expectation as “what is predicated to occur rather than what is desired.” According to expectation violation theory (Burgoon, 1993), an individual interacts with others with expectancy that refers to what will happen in a given situation and so, the disconfirmation of such expectancy negatively influences outcomes of the interaction. In the hospitality service context, customers seem to have a certain level of *emotional service expectation* and want to confirm it during the interaction; they expect employees to express positive emotions, such as friendliness, positivity, compassion, and/or warmth, while suppressing negative emotions, such as anger, indifference, or frustration (Beal et al., 2006; Grandey and Gabriel, 2015).

The literature has suggested that customer expectation positively influences confirmation. For example, Park et al. (2018) argued that high expectation increases customers’ involvement levels, leading to easy confirmation. Groth et al. (2009) also empirically demonstrated that positive attitude with high expectation results in high confirmation. Consistent with the results in the existing studies, this study hypothesized that customers’ expectation of emotional services will positively influence overall confirmation of a service outcome.

***H1:*** *Emotional service expectation has a positive effect on overall confirmation.*

### Emotional Service Expectation Toward Perceived Quality and Customer Satisfaction

In their seminal work on SERVQUAL, Parasuraman et al. (1985) indicated that *service quality* is determined by the evaluation of a service provider by comparing the service provider’s performance with the customer’s *expectations* of how similar service providers should perform. In other words, customers set the range of expected outcomes of emotional services as a quality standard in which a given service quality is likely to be evaluated (Tsai, 2001; Humphrey et al., 2007). Therefore, quality of services depends on customers’ expectation of how effectively and positively employees express positive emotions. In addition to service expectation, *overall confirmation* is likely to directly influence quality perception. Parasuraman et al. (1988) stated that service quality results from overall assessments in an integral dimension of expectations. Grönroos (1984) addressed service quality can be measured in the two dimensions of process quality and technical quality, and these two dimensions are intricately connected, indicating service quality is related to overall confirmation. Confirming this argument, Homburg and Stock (2004) argued that quality perception is related to customers’ confirmation of cognitive and affective factors of services. In a hotel setting, satisfying interaction experiences with employees confirmed customer expectation of emotional services and so, enhanced overall service quality perception (Ladhari, 2009). Further, Brady and Cronin (2001) demonstrated quality perception is determined by overall impression of a given service. Since the nature of service involves intangibility and inseparability, service quality seems to be influenced by overall confirmation of expectations. In summary, the literature suggested that perceived quality is influenced by customer expectation and overall confirmation, so we hypothesized as follows:

***H2****: Emotional service expectations have a positive effect on perceived quality.*
***H3:*** *Overall confirmation has a positive effect on perceived quality.*

Gracia et al. (2011) found that five service quality components, including reliability, assurance, responsiveness, empathy, and tangibles, have positive effects on customer emotions and, as a result, increase satisfaction in the hotel and restaurant service context. Han and Jeong (2013) also empirically demonstrated that customers’ service quality perception significantly influences service experiences and emotional satisfaction. Due to their close relationship, quality perception is often used as an indicator of measures of customer satisfaction (Homburg and Stock, 2004; Wong, 2004).

Ladhari (2009) claimed that satisfaction consists of cognitive (i.e., customer’s judgment) and affective components (i.e., happy, pleasant, and joyful), both of which are evaluated to confirm service expectation. In addition, customer satisfaction seems to be influenced by various external attributes, such as in-store environment and feelings (Han, 2013). Therefore, this study hypothesized that both perceived quality and overall confirmation influence customer satisfaction.

***H4****: Perceived quality has a positive effect on customer satisfaction.*
***H5****: Overall confirmation has a positive effect on customer satisfaction.*

### Two Emotional Strategies: Deep and Surface Acting

Emotional services, such as displays of enthusiasm, friendless, and warmth, are identified as the important services of worth in service delivery (Diefendorff et al., 2006; Sutton et al., 2013). The literature has confirmed that emotional services have positive effects on customer perceptions and satisfaction (Groth et al., 2009; Grandey and Gabriel, 2015). Lin and Liang (2011) study also confirmed that displays of positive emotions increase customers’ positive responses, such as customer delight, repurchase intent, and positive word-of-mouth. Similarly, Johanson and Woods (2008) argued that emotional services help achieve organizational goals, such as customer satisfaction and firms’ long-term profitability.

Service firms, in general, require employees to regulate their emotions to express only positive emotions to customers. To comply with the job requirement, employees tend to adopt two main strategies of emotional displays: deep acting and surface acting. Deep acting refers to “good faith” by “putting one’s self in another’s shoes” (Diefendorff et al., 2006) and it is a genuine feeling created within themselves (Hennig-Thurau et al., 2006). Deep acting is regarded as sincerely showing the emotions that match their genuine feelings and organization desires by feeling customers’ feelings and having empathy (Grandey, 2003). In turn, employees who use deep acting are likely to be customer-oriented and so, provide their customers sincere service (Allen et al., 2010). Deep acting has been reported to increase job performance and, as a result, lead to positive feedback from customers (Hatfield et al., 1994). In contrast, surface acting involves “simulating emotions that are not actually felt” (Ashforth and Humphrey, 1993, p. 92). Surface acting occurs when service employees modify only their visible emotions and deceive customers by “putting on a mask,” without actually changing how they feel. Surface acting can bring out an emotional discrepancy between true feelings and expressions because employees just pretend to feel positive to meet the organization’s requirements (Johnson and Spector, 2007).

### The Moderating Role of Employee Deep/Surface Acting

Mattila and Enz (2002) stated that customers’ evaluation of service consumption experience depends on how effectively employees display positive emotions during customer interactions. Deep acting strategy allows employees to express their genuine feelings in line with the desired emotions that service firms require, and customers expect (Diefendorff and Greguras, 2009). Deep acting is related to trustworthiness and authenticity that have been known as the main components of service performance to enhance quality perceptions and satisfaction (Krumhuber et al., 2007). Indeed, employees who use deep acting are likely to understand customers and respond to their needs well (Sandström et al., 2008).

When employees cannot modify their inner feelings, employees may use the surface acting strategy (Diefendorff and Greguras, 2009), but this may result in negative outcomes (Hatfield et al., 1994). Grandey et al. (2005) stated “when service providers do not seem sincere in their expressions… it is less likely to create a positive impression in the customer; instead, a false smile may seem manipulative and the employee’s impression management attempt fails” (p. 52). Since people tend to prefer honesty and authenticity in social interactions, employees’ fake emotional displays are unlikely to meet customers’ emotional expectation (Grandey, 2003; Hennig-Thurau et al., 2006). From the employees’ perspective, surface acting may also impair their service performance in that expressing a fake emotion involves additional waste of cognitive resources, affecting job satisfaction (Johnson and Spector, 2007). According to fit theory, when there is wide discrepancy between outward and inward feelings at work, employees are likely to be frustrated and dissatisfied with their jobs, decreasing work performance (Gabriel et al., 2015) which is a primary reason of negative feedback from customers (Heskett et al., 1994).

**FIGURE 1:**
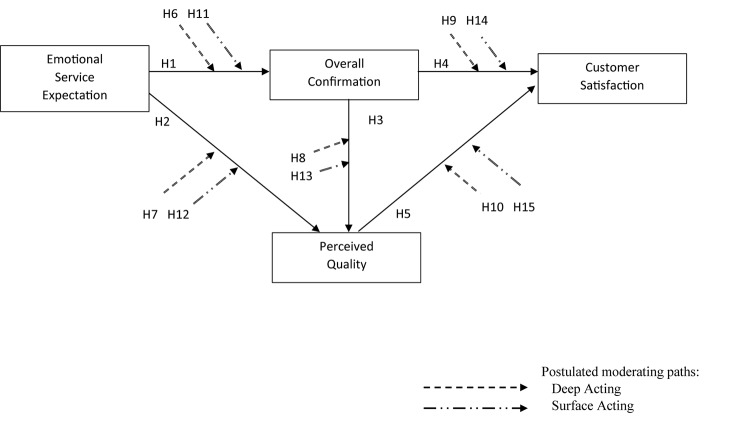
Theoretical model paths.

All things considered, it was anticipated detecting deep acting is likely to strengthen the link of expectation-confirmation toward satisfaction, while detections of surface acting may counteract it. In this study, Hypotheses 6 through 10 were proposed with regard to the positive moderating effects of customer detection of deep acting while Hypotheses 11 through 15 were about the negative moderating effects of customer detection of surface acting. All hypothesized paths in the study model are shown in Figure 1.

***H6–10:*** *Customer detection of deep acting positively moderates the relationship among the study constructs (i.e., emotional service expectation, overall confirmation, perceived quality, and satisfaction).*
***H11–15****: Customer detection of surface acting negatively moderates the relationship among the study constructs (i.e., emotional service expectation, overall confirmation, perceived quality, and satisfaction).*

## Materials and Methods

### Participants and Procedure

A web-based online survey using email invitation was conducted using a nationally recognized consumer research panel service. A sample of 220 individuals from across the United States over 20 years old was used with screening questions of their hotel service experience with scenario explanation. All participants who satisfied the sampling conditions, opted in to the survey in exchange for a credit of $1. Participants answered the questions to examine the role of emotional service expectation toward customer satisfaction and the moderating roles of detections of deep acting and surface acting on their relationships in the service industry. The measurement items were adopted based on the literature review. Table 1 shows demographic results, including gender, age, income, marital status, education level, and annual income. Males made up 51.4% and 48.6% was female. Participants were categorized by age into young adults (ages 20–39 years; *n* = 142) and older adults (ages 40–50 and over+ years, *n* = 78). More than half of the sample had less than $50,000 in annual income.

**Table 1:** Respondents’ socio-demographic characteristics.

Variable	Group	Frequency	Percent
**Age**	20–29	51	23.2
	30–39	91	41.4
	40–49	37	16.8
	50 and over	41	18.6
	*Total*	*220*	*100*.*0*
**Gender**	Male	113	51.4
	Female	107	48.6
	*Total*	*220*	*100*.*0*
**Income**	Under $20,000	33	15.0
	20,001–30,000	45	20.5
	30,001–40,000	46	20.9
	40,001–50,000	27	12.3
	60,001–70,000	24	10.9
	70,000 or over	45	20.5
	*Total*	*220*	*100*.*0*
**Marital**	1	104	47.3
	2	16	7.3
	3	5	2.3
	4	95	43.2
	*Total*	*220*	*100*.*0*
**Education**	High school	27	12.3
	2-year college	72	32.7
	Bachelor’s degree	84	38.2
	Graduate school	33	15.0
	High school	4	1.8
	*Total*	*220*	*100*.*0*


### Measures

The questionnaires contained question items focusing on the emotional service expectation, overall confirmation, perceived quality, customer satisfaction, and customer detection of deep and surface acting. Latent variables are not directly observable, so compound multi-item scale measures, including at least two items for each variable, were used (Kenny, 2014). Based on the existing literature, well-validated measurement items for study constructs were adopted and included in the questionnaire. Specifically, emotional service expectation measures were adopted from Davis et al. (1999) three-item scale using sympathy, enjoyment, and compassion dimensions. Overall confirmation was assessed using a two-item scale developed by Oliver (1980). For perceived service quality, three items were adopted from Fornell and Larcker (1981) study. A 5-point Likert-type scale was used, with response options ranging from 1 to 5 in three different dimension standards (e.g., 1 = “poor,” 5 = “excellent”; 1 = “inferior,” 5 = “superior”; and 1 = “low,” 5 = “high”). For customer satisfaction, two items were borrowed from Westbrook and Oliver (1991) study, designed to measure customer satisfaction and emotions with services. To assess customer detections of employees’ deep and surface acting, three-item measures were derived from previous study (Groth et al., 2009). These measures were developed “from Grandey (2003), originally developed by Brotheridge and Lee (2003)” (Groth et al., 2009, p. 964). Given the fact that most recent studies still adopt the measures of Brotheridge and Lee (2003) or Grandey (2003), Groth et al. (2009) measures are relatively updated (see Baranik et al., 2017; Uy et al., 2017; Lam et al., 2018; Moin, 2018). Except for quality dimensions, all other variables consistently used the same semantic measurement tool (e.g., 1 = “I would not feel this way at all,” 5 = “I would feel this way very much”). All items included in each variable are reported in Table 2.

**Table 2:** Items, standardized factor loadings and Cronbach’s α.

Constructs/Scale items	Standardized factor loadings	Cronbach’s α
**Emotional service expectation**		0.789
I anticipated experiencing sympathy in relation to this service provider.	0.813	
I anticipated experiencing compassion in relation to this service provider.	0.952	
I anticipated experiencing enjoyment in relation to this service provider.	0.510	
**Overall confirmation**		0.815
Overall, this service was worse than expected.^1^	0.805	
Overall, this service was better than expected.	0.871	
**Perceived quality**		0.975
Poor–excellent	0.969	
Inferior–superior	0.955	
Low standards–high standards	0.968	
**Customer satisfaction**		0.933
I am satisfied with my decision to visit this service provider. I think I did the right thing when I purchased this service.	0.979 0.893	
**Deep acting emotional labor**		0.934
This service provider tried to actually experience the emotions s/he had to show to me.	0.914	
This service provider worked hard to feel the emotions that s/he needed to show to me.	0.882	
This service provider made a strong effort to actually feel the emotions that s/he needed to display toward me.	0.931	
**Surface acting emotional labor**		0.898
This service provider just pretended to have the emotions s/he displayed to me.	0.871	
This service provider put on a ‘mast’ in order to display the emotions his/her boss wants him/her to display.	0.918	
This service provider showed feelings to me that are different from what s/he actually felt.	0.805	


*^*1*^Reverse coded item*.

## Results

### Measurement Model Tests

Before analyzing the structural model, the measurement model was assessed with Confirmatory Factor Analyses (CFA) using AMOS 22 and SPSS. CFA evaluated goodness of fit with the six key variables, including emotional expectations, conformation, quality perception, satisfaction, detections of deep acting and surface acting. The results confirmed good fit indices (χ^2^_86_ = 175.408, *p* < 0.000; RMSEA = 0.063; CFI = 0.978; NFI = 0.959; GFI = 0.925; RFI = 0.942; IFI = 0.978; TLI = 0.970) of the measurement model. Factor loadings for the indicators for each variable were all significant and sufficiently higher than the recommended value of 0.50 (Table 2), indicating convergent validity (Grayson and Marsh, 1994). All Cronbach’s alpha values for each variable were above the minimum threshold of 0.70 (Hair et al., 1998), suggesting internal consistency in measurement items. As shown in Table 3, composite reliability values for each construct ranged from 0.815 to 0.975, greater than 0.70, indicating good reliability (Hair et al., 2011). Average value extracted (AVE) values ranged from 0.609 to 0.929, all above 0.50, confirming convergent validity (Hair et al., 2011). Also, these AVE values were all greater than the square of correlation between pairs of constructs, achieving discriminant validity (Fornell and Larcker, 1981). All constructs’ correlations were less than the threshold of 0.85, confirming further discriminant validity (Kline, 2005). The means, standard deviations, composite reliability, average variance extracted, and Pearson’s correlations of variables are reported in Table 3.

**Table 3:** Means, standard deviations, reliabilities, AVE, and correlations.

	DA	SA	ESE	OC	PQ	CS
DA	0.935^a^	0.020	0.141	0.460	0.364	0.476
SA	-0.141^b^	0.900	0.011	0.075	0.044	0.032
ESE	0.375	0.107	0.815	0.132	0.125	0.163
OC	0.678	-0.273	0.363	0.826	0.630	0.672
PQ	0.603	-0.209	0.354	0.794	0.975	0.601
CS	0.690	-0.180	0.404	0.820	0.775	0.931
Means	4.239	3.813	4.232	4.333	4.442	4.693
SD	1.510	1.490	1.290	1.702	1.680	1.584
AVE	0.827	0.750	0.609	0.703	0.929	0.878


*DA, deep acting; SA, surface acting; ESE, emotional service expectation; OC, overall confirmation; PQ, perceived quality; CS, customer satisfaction; SD, standard deviation; AVE, average variance extracted. Model measurement fit: χ^2^_*86*_ = 175.408, *p* < 0.000; RMSEA = 0.063; CFI = 0.978; NFI = 0.959; GFI = 0.925; RFI = 0.942; IFI = 0.978; TLI = 0.970. ^*a*^Composite reliabilities highlighted in shade are along the diagonal. ^*b*^Correlations between constructs are below the diagonal. ^*c*^Squared correlations between constructs are above the diagonal.*

### Structural Model Tests

The results of the structural equation modeling (SEM) with maximum likelihood estimation procedure showed a good model fit (χ^2^_25_= 53.801, *p* < 0.000; χ^2^/df = 2.151; GFI = 0.961; NFI = 0.980; CFI = 0.989; RFI = 0.964; IFI = 0.989; TLI = 0.981; RMSEA = 0.066). Table 4 presents empirical findings of the hypothesized relationships within the original model. Emotional service expectations were found to significantly and positively influence the overall confirmation (β = 0.582, *p* < 0.001, H1), but not significantly influence perceived quality (β = -0.054, *p* = 0.401, H2). Overall confirmation significantly and positively linked to perceived quality (β = 0.899, *p* < 0.001, H3) and customer satisfaction (β = 0.930, *p* < 0.001, H4). The hypothesized path from perceived quality to customer satisfaction was found to be insignificant (β = 0.004, *p* = 0.973, H5). Overall, the findings supported Hypotheses 1, 3, and 4, but provided no support for Hypotheses 2 and 4.

**Table 4:** Results of the tests of path coefficients.

Hypotheses	Paths	Path coefficient (β)	*p*-value
H1 (supported)	ESE → OC	0.582	^∗∗∗^
H2 (rejected)	ESE → PQ	-0.054	0.401
H3 (supported)	OC → PQ	0.899	^∗∗∗^
H4 (supported)	OC → CS	0.930	^∗∗∗^
H5 (rejected)	PQ → CS	0.004	0.973


*^∗∗∗^*p* < 0.001, ^∗∗^*p* < 0.01, ^∗^*p* < 0.05. Goodness-of-fit: χ^*2*^_*25*_ = 53.801, *p* < 0.000; GFI = 0.961; NFI = 0.980; CFI = 0.989; RFI = 0.964; IFI = 0.989; TLI = 0.981; RMSEA = 0.066. ESE, emotional service expectation; OC, overall confirmation; PQ, perceived quality; CS, customer satisfaction.*

### Tests of Measurement Invariance

Prior to testing moderating effects, a measurement invariance test across two groups of moderators was conducted (Steenkamp and Baumgartner, 1998). In this study, respondents were divided into two groups of each of moderators of deep and surface acting by a median-split method: high (*n* = 130) and low (*n* = 90) groups of deep acting; and high (*n* = 113) and low (*n* = 107) groups of surface acting. For each moderator, an unconstrained model (baseline model) and a constrained model (invariance model) were generated and tested. In the invariance model, factor loading, factor variances, and covariances were constrained to be equivalent across the two groups from the baseline model. As indicated in Table 5, Fit indices of Root Mean Square Error of Approximation (RMSEA) and Comparative Fit Index (CFI) were checked because they are relatively less sensitive to sample size (Fan et al., 1999), while the Chi-square index is relatively sensitive to sample size (Byrne, 2001).

**Table 5:** Results of measurement invariance test.

		χ^2^	df	CFI	RMSEA	Δχ^2^/df	Δχ^2^ Sig. df
Deep acting	Baseline modelccc (unconstrained)	125.213	52	0.966	0.073	9.481/6	No
	Invariance modelccc (λ constrained)	134.694	58	0.965	0.071		
Surface acting	Baseline modelccc (unconstrained)	118.909	52	0.976	0.070	4.362/6	No
	Invariance modelccc (λ constrained)	123.271	58	0.976	0.066		


With regard to *deep acting*, the overall model fits were excellent both for the baseline model (χ^2^_52_= 125.213, CFI = 0.966; RMSEA = 0.073) and the invariance model (χ^2^_58_= 134.694, CFI = 0.965; RMSEA = 0.071) (Table 5). The difference in χ^2^ between those two models was insignificant (Δχ^2^_6_ = 1.580, *p* > 0.05), supporting the invariance model. The results confirmed that the measurement model was equivalent across high and low groups, so the invariance model was employed for the subsequent analyses of moderating effects of deep acting.

The structural invariance test for *surface acting* were conducted in the same way; the baseline (unconstrained) model (χ^2^_52_ = 118.909, CFI = 0.976; RMSEA = 0.070) and the invariance (constrained) model (χ^2^_58_ = 123.271, CFI = 0.976; RMSEA = 0.066) showed excellent fits to the data. Again, there was no significant difference in χ^2^ between the baseline and invariance models (Δχ^2^_6_ = 0.702, *p* > 0.05), supporting the invariance model. Therefore, the invariance model was adopted for subsequent analysis of moderating effects of surface acting.

### Tests of Moderating Effect of Deep and Surface Acting

**FIGURE 2:**
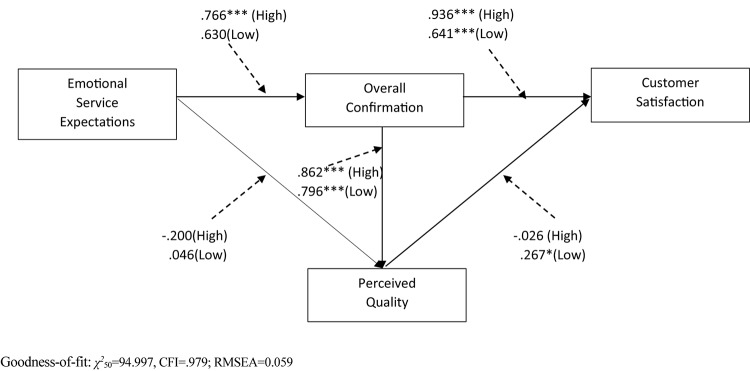
Results of the structural invariance model with deep acting. Dash lines indicate hypothesized paths of moderating impact of deep acting. ^∗∗∗^*p* < 0.001, ^∗∗^*p* < 0.01, ^∗^*p* < 0.05.

For the moderating effect tests of *deep acting*, a baseline model was generated by adding the hypothesized links among the variables based on the invariance model in both high and low deep acting groups. As shown in Table 6, the baseline model satisfactorily fit to the data (χ^2^_50_ = 94.997, *p* < 0.001, CFI = 0.979; RMSEA = 0.059). In the nested models, the hypothesized paths were constrained to be equal for high and low groups. The baseline model and a series of the nested models (equal path model) were compared in pairs to analyze moderating effects of deep acting. Contrary to our expectations, the differences in χ^2^ between high and low deep acting groups on each of the hypothesized paths were all found to be insignificant, rejecting Hypotheses 6, 7, 8, 9, and 10 (Figure 2 and Table 6). In other words, the original relationships among the variables were not moderated by deep acting. The findings related to deep acting were reported in Figure 2 and Table 6.

**Table 6:** Results of path and model comparison of deep acting.

								Measurement weights of					
		Deep acting: high			Deep acting: low			nested models (λ constrained)					
		Path	Critical		Path	Critical							Δχ^2^
	Paths	coefficient	ratio (*t*-value)	*p*-value	coefficient	ratio (*t*-value)	*p*-value	χ^2^	df	CFI	RMSEA	Δχ^2^/df^1^	Sig. df
H6	ESE → OC	0.766	4.375	^∗∗∗^	0.630	1.537	0.124	96.894	51	0.979	0.059	1.897/1	No
H7	ESE → PQ	-0.200	-1.037	0.300	0.046	1.769	0.077	98.314	51	0.978	0.060	3.317/1	No
H8	OC → PQ	0.862	4.075	^∗∗∗^	0.796	8.895	^∗∗∗^	96.045	51	0.979	0.058	1.048/1	No
H9	OC → CS	0.936	5.852	^∗∗∗^	0.641	4.516	^∗∗∗^	97.808	51	0.979	0.059	2.811/1	No
H10	PQ → CS	-0.026	-0.247	0.805	0.267	2.106	0.035^∗^	97.744	51	0.979	0.059	2.747/1	No


*^∗∗∗^*p* < 0.001, ^∗∗^*p* < 0.01, ^∗^*p* < 0.05. ^*1*^Each of the nested models was compared with a baseline model (unconstrained): χ^*2*^_*50*_ = 94.997, CFI = 0.979; RMSEA = 0.059.*

**FIGURE 3:**
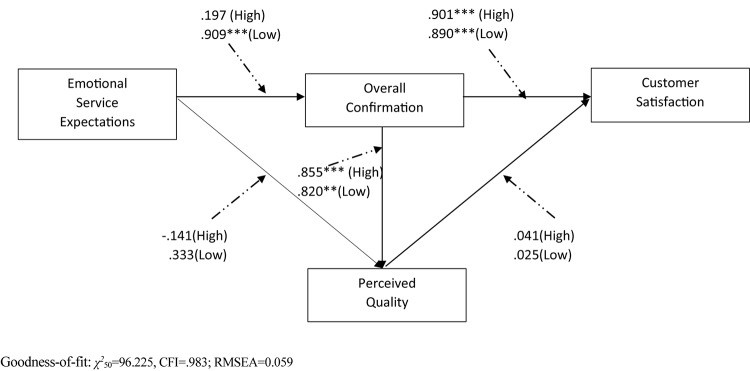
Results of the structural invariance model with surface acting. Dash lines indicate hypothesized paths of moderating impact of surface acting. ^∗∗∗^*p* < 0.001, ^∗∗^*p* < 0.01, ^∗^*p* < 0.05.

In the same way, a baseline model and a series of nested models (equal path model) for *surface acting* were compared in pairs (Table 7), and the baseline model provided an excellent fit to the data (χ^2^_50_ = 96.225, CFI = 0.983; RMSEA = 0.059). On the path from emotional service expectation to overall confirmation, there was a significant difference in Chi-square between the baseline model and the nested model (χ^2^_51_ = 112.931, CFI = 0.978; RMSEA = 0.068, Δχ^2^_1_ = 16.706, *p* < 0.001), in support of Hypothesis 11. The positive effect of emotional service expectation on overall confirmation was significant when surface acting was *less* detected (β = 0.909, *t*-value = 5.677, *p* < 0.001), while such a positive effect turned to be insignificant when surface acting was strongly detected (β = 0.197, *t*-value = 1.258, *p* = 0.208). That means that surface acting offsets the positive influence of emotional service expectation on overall confirmation. The other nested models were not significantly different from the baseline model, rejecting Hypotheses 12, 13, 14, and 15. The findings of path model comparisons for surface acting were reported in Figure 3 and Table 7.

**Table 7:** Results of path and model comparison of surface acting.

								Measurement weights of					
		Surface acting: high			Surface acting: low			nested models (λ constrained)					
		Path	Critical		Path	Critical							Δχ^2^
	Paths	coefficient	ratio (*t*-value)	*p*-value	coefficient	ratio (*t*-value)	*p*-value	χ^2^	df	CFI	RMSEA	Δχ^2^/df^1^	Sig. df
H11	ESE → OC	0.197	1.258	0.208	0.909	5.677	^∗∗∗^	112.931	51	0.978	0.068	16.706/1	Yes
H12	ESE → PQ	-0.141	-1.257	0.209	0.087	0.333	0.739	96.565	51	0.983	0.058	0.340/1	No
H13	OC → PQ	0.855	8.809	^∗∗∗^	0.820	3.210	0.001^∗∗^	97.047	51	0.983	0.059	0.822/1	No
H14	OC → CS	0.901	5.733	^∗∗∗^	0.890	5.379	^∗∗∗^	97.027	51	0.983	0.059	0.802/1	No
H15	PQ → CS	0.041	0.312	0.755	0.025	0.163	0.870	96.230	51	0.984	0.058	0.004/1	No


*^∗∗∗^*p* < 0.001, ^∗∗^*p* < 0.01. ^*1*^ Each of the nested models was compared with a baseline model (unconstrained): χ^*2*^_*50*_ = 96.225, CFI = 0.983; RMSEA = 0.059.*

## Discussion

This study aimed to develop an alternative conceptual model articulating the role of *emotional service expectation* toward customer satisfaction and the moderating roles of customer detections of deep acting and surface acting on their relationships; the five paths were proposed among the variables, including emotional service expectation, overall confirmation, perceived quality, and customer satisfaction, and then detections of deep and surface acting were imposed on each of the paths to verify the moderating effects. We hypothesized that the detection of deep acting would cement each of the relationships while the detection of surface acting would weaken those relationships.

As expected, the role of customer’s emotional service expectation was significant in overall service confirmation (Hypothesis 1), which further influenced perceived quality (Hypothesis 3) and satisfaction (Hypothesis 4). The results are consistent with expectation violation theory (Burgoon, 1993) positing that an individual interacts with others with expectancy in which interaction outcomes are determined. Also, the study’s results supported Farrell and Oczkowski (2009) arguments that service outcomes are likely to be confirmed by employee–customer interactions that take place for service delivery. However, this study did not find the significant relationship between perceived quality and customer satisfaction (Hypothesis 5); this is not congruent with the dominant belief in existing studies. We postulate, overall confirmation sufficiently strongly influences customer satisfaction (Hypothesis 4), so the influence of perceived quality on customer satisfaction becomes relatively weak in this study construct.

A growing body of research has demonstrated the roles of emotional strategies in customer judgments and perceptions (Groth et al., 2009; Hülsheger et al., 2010; Grandey and Gabriel, 2015). Hülsheger et al. (2010) suggested that influences of emotional strategies on the customer’s decisions are significant and universal. However, in this study, the moderating effects of customer detection of emotional strategies were limited to the influence of emotional service expectation on overall confirmation; Customer detection of employees’ surface acting negatively influences service confirmation in the beginning stage of interaction (Hypothesis 11) and, as a result, indirectly decreases perceived quality and satisfaction. Whereas all the other hypothesized moderating effects were found to be insignificant. Especially, detections of deep acting hardly influence customer perception and evaluation of services. These results are somewhat consistent with Hur et al. (2015), indicating there were no direct impacts of employees’ deep acting strategies on service assessments.

### Theoretical and Practical Implications

Despite a growing awareness of the critical roles of employees’ emotional services, little has been known about how customers’ emotional service expectations and employees’ emotional displays influence service assessments in the hospitality service context. The major theoretical contribution of this study demonstrates the role of employees’ emotional display strategies in the relationship between customer emotional service expectation and overall confirmation that further influenced satisfaction and perceived quality. This study extends the well-known body of literature articulating the roles of expectation in customer satisfaction (Oliver, 1980; Teas, 1993) by integrating emotional factors in a manner in which customer emotional expectation-confirmation influences perceived quality and satisfaction that are moderated by emotional display strategies. This study provides confirmative evidence that emotional service expectation and emotional display strategies play a critical role in service confirmation, suggesting that comprehensive understating of customer behaviors in the hotel service context is critical.

Also, this study successfully extends the traditional model framework consisting of direct and mediating effects based on expectancy-disconfirmation theory by incorporating emotional components as moderators. There are two contrasting theories explaining the effects of expectations. *Direct effect hypothesis* claims that expectations have a direct, positive impact on customer satisfaction (Park et al., 2018) while *Affective expectation model* emphasizes roles of discrepancy between actual and expected service outcomes in satisfaction (Wilson and Klaaren, 1992). The results of this study confirmed both of the expectation theories. Emotional service expectation has direct, positive effects on service outcomes (H1) while its influences on overall confirmation differ by detections of high and low surface acting (H11).

Customers have typical expectancies about employees’ emotional services, such as “service with a smile” (Parasuraman et al., 1985; Schminke et al., 2014). Burgoon and Walther (1990) claimed that expectancy is associated with predictions rather than desires. Consistent with their arguments, customers seem to *predict authentic emotional services* from employees; surface acting such as faking smiles appears not enough to enhance positive outcomes and even worse, has negative impacts on overall confirmation. Service managers are “wise to want workers to be sincere, to go well beyond the smile that’s just painted on” (Hochschild, 1983, p. 33). Even though some researchers argued that there is no difference in customer reactions and service outcomes between displays of authentic and fake emotions (Beal et al., 2006; Chi et al., 2011; Wang and Groth, 2014), the study’s results suggest that customer decisions can differ by customers’ detections of employees’ emotional displays, whether they are real or fake. Thus, service organizations need to intervene to help their employees adopt deep acting strategies to meet customer emotional service expectations. Possible training may include perspective taking and empathy training to help employees understand customer’s perspectives and display genuine emotions. In summary, managerial emphasis should be on hiring those who can genuinely express their emotions and training them to understand customer’s needs sincerely (Parker and Axtell, 2001).

### Limitation and Future Research

As is the case with any research, there are limitations that can be suggested for future research. First, this study explored the influence of customers’ *predictive* expectations as an independent variable, so the other dimensions of expectations need to be investigated. Expectations can be classified into normative (i.e., what should be), predictive (i.e., what customers really expect), and equitable expectations (i.e., what customers should receive, taking into account the expenses borne) (Medrano et al., 2016). For the future research, it seems worthwhile comparing influences of different dimensions of customers’ expectations. Second, this study used an online panel in which respondents were asked questions recalling their most recent service experiences. While the use of an online panel is suitable to collect a large sample and the use of experience and memory based measures of emotions allows researchers to investigate real service users, these methods are subject to several response biases, such as selection bias, non-response bias, recall bias, and memory bias as questionnaires are not distributed perfectly randomly and/or participants have difficulty in retrieving the detailed information of the past experience. Another limit of this study is that the questionnaires were collected in the United States, and there may be different findings across cultures and nations. Norms and interpretations about emotion-communication may be specific to cultures, so customer responses to employees’ emotional displays are likely to vary across cultures (Triandis, 1989). For example, compared with individualist cultures, collectivist cultures tend to emphasize connectedness to other people, conformity, and social contribution (Ariely, 2008), so more severe emotional duties and obligations are likely to be implicitly imposed on employees in these cultures. In turn, customers in collectivist cultures may strongly predict employees’ deep acting strategies, while being more sensitive to surface acting. Fourth, factors unrelated services, such as weather, personality and customer emotions *per se*, may influence customers’ assessment of employees’ emotion displays (Penz and Hogg, 2011; Pelegrín-Borondo et al., 2015). Therefore, more properly controlled experiments seem to be necessary for future research to clearly determine causality among the variables. Lastly, it is necessary to explore which categorical emotional displays are responsible for the degree of customers’ detections of deep and surface action (Pelegrín-Borondo et al., 2015). Then, future research may give even greater insights into human resource management practically and academically in the hospitality industry.

## Author Contributions

All authors listed have made a substantial, direct and intellectual contribution to the work, and approved it for publication.

## Conflict of Interest Statement

The authors declare that the research was conducted in the absence of any commercial or financial relationships that could be construed as a potential conflict of interest.
